# Role model narratives are underutilized in Instagram and TikTok climate action posts

**DOI:** 10.1093/oxfclm/kgaf028

**Published:** 2025-11-19

**Authors:** Julia Fine, Joshua Ettinger, Sri Saahitya Uppalapati, John Kotcher, Edward Maibach

**Affiliations:** Center for Climate Change Communication, George Mason University, Fairfax, VA 22030, United States; Center for Climate Change Communication, George Mason University, Fairfax, VA 22030, United States; Center for Climate Change Communication, George Mason University, Fairfax, VA 22030, United States; Natural Resources Defense Council, Washington, DC, United States; Center for Climate Change Communication, George Mason University, Fairfax, VA 22030, United States; Center for Climate Change Communication, George Mason University, Fairfax, VA 22030, United States

**Keywords:** climate change, role models, narrative, social media, communication

## Abstract

Role model narratives—stories about specific people taking recommended actions—are a proven communication strategy for encouraging socially beneficial behaviors such as climate action. However, the extent to which climate activists share role model narratives on social media has yet to be investigated. To measure the prevalence of climate action role model narratives on social media, we manually collected Instagram and TikTok posts tagged with the hashtags #climateaction and #climateactivist (dated between August 2021 and October 2024) and conducted a content analysis of the posts (Instagram *N* = 457 and TikTok *N* = 188). We examined the types of actions featured in role model narratives, the presence and nature of any calls to action, and whether role model narratives are correlated with higher engagement than other types of content. We found that role model narratives appear in only 11% of the Instagram posts (*N* = 48) and 19% of the TikTok posts (*N* = 35), which suggests that they are an underutilized communication approach. Furthermore, only 28% of the role model narratives included calls to action, with joining an organization being the most common. Additionally, role model narratives did not have significantly different engagement compared to other posts in the sample, but were represented among the 10 top-performing posts. Given the ease with which the role model narrative format can be adopted and the powerful reach of social media, climate groups and advocates have a unique opportunity to share their stories and lead by example, using their platforms to inspire greater and more concerted climate action.

## Introduction

About one in four Americans view climate change as an urgent threat and strongly support climate action [1]. However, most are not taking action to advocate for climate solutions. Only 34% of those who are alarmed are actively engaging in climate activism, although 46% say they would be willing to, and 20% are uncertain whether they would [2]. The disparity between people’s climate change concerns and actions is known as the environmental attitude-behavior gap [3]. Numerous studies have sought to understand the barriers to these behaviors, including the perceived individual or collective efficacy of particular behaviors, social norms, and real or perceived costs, among other factors [4]. The stakes of overcoming these barriers are especially high: collective public engagement is essential to building the political will and public will necessary for ambitious climate policy [5]. When large numbers of the public mobilize through voting, contacting elected officials, protesting, or supporting pro-climate policies, they can shift policy agendas and pressure policymakers to act on climate change. Therefore, understanding what motivates or inhibits public participation in climate advocacy is critical to achieving broad and durable climate solutions [6].

Effective climate communication strategies can play a crucial role in addressing the attitude–behavior gap by making the issue more salient, connecting climate impacts to people’s everyday lives, and framing action as both necessary and achievable. One promising approach is the use of role model narratives, also known as behavioral journalism. Grounded in Bandura’s (1986) social cognitive theory, role model narratives aim to teach people by example about an action worth taking, how to take the action, and the benefits of taking action, thereby increasing audience members’ self-efficacy to perform the behavior and outcome expectancies that doing so will be beneficial [7, 8]. Through observational learning, audiences see how others engage in certain actions, including the challenges they face and overcome. This provides a blueprint for their own behavior [9]. Furthermore, reciprocal determinism—the dynamic interaction between personal factors, behavior, and the environment—is activated as these narratives circulate within social networks, influencing social norms, identity, and further engagement [7]. In the case of climate change, role model narratives have the potential to make climate engagement feel accessible and realistic by showcasing people “like me” taking meaningful steps.

This concept has a long history as a successful communication strategy for health behaviors, such as efforts to reduce community-level HIV risks and to improve dietary choices and physical exercise among low-income individuals [10, 11]. Although climate communication researchers have largely overlooked this approach, Kotcher *et al*. (2024) found that when health professionals received role model narratives about climate action via an email newsletter, they increased their perception that healthcare professionals have a responsibility to act on climate change, as well as their willingness to receive information on how to join a climate and health advocacy group [12].

Given the above evidence, role model narratives are a potentially promising strategy for encouraging increased adoption of climate-related behaviors. Compared to commonly used climate communication strategies, such as informational approaches and fear appeals, role model narratives may offer unique advantages. Informational messaging often emphasizes facts and scientific evidence to convey the urgency of climate change [13]. While these approaches can enhance knowledge, they are frequently perceived as abstract and complex, which may increase psychological distance and limit their effectiveness in motivating behavior change [14–16]. Similarly, fear appeals that seek to evoke concern or urgency through emotive messaging and vivid depictions of climate impacts may backfire by inducing feelings of helplessness or avoidance if not accompanied by clear efficacy cues [17–19]. In contrast, role model narratives offer a more accessible and action-oriented alternative. They are straightforward to create, requiring no special skills; at their most basic level, they are merely a demonstration of a recommended action—by the storyteller or someone else—that should produce a desired outcome.

Role models are ubiquitous on social media, for instance in social media influencers’ promotion of products [20]. Social media platforms are important communicative spaces for encouraging dialogue about climate change, as well as building awareness, networks, and mobilizing action [21]. Recent health behavior research suggests that social media role models can affect their audiences’ health behavior motivations [22]. Furthermore, recent literature suggests that environmental communicators employ climate action role model narratives on social media. San Cornelio, Martorell, and Ardèvol (2024) found that eco-influencers on Instagram seek to encourage individual climate action by using first-person narratives combined with positive emotions and humor, which make posts more engaging [23]. Similarly, Haastrup and Marshall (2024) describe how climate influencers on Instagram often showcase their own pro-environmental behaviors to encourage their followers to also pursue sustainable lifestyle choices [24]. Interviews with followers of these influencers suggest followers perceive a resemblance between their own lives and the influencers’, and that this perceived resemblance may help encourage the adoption of role model behaviors [25]. However, the incidence of climate action role model narratives on social media, with respect to other types of content posted by environmental communicators, remains unknown.

In this study, we conduct a content analysis to examine the presence and characteristics of climate action role model narratives on two widely used social media platforms. We focus on Instagram and TikTok as they are among the most commonly used social media platforms worldwide, are publicly accessible, and are known to be sites of activity for climate change storytellers [26, 27]. TikTok has emerged recently as a particularly popular platform for individual creators and influencers passionate about environmental issues [27, 28]. For the purposes of this study, we define climate role model narratives as social media posts that showcase one or more individuals’ climate-related behaviors in an effort to encourage others to pursue similar behaviors. This study builds on prior literature by offering new insights into the incidence and content of climate action role model narratives on social media. Our research questions were as follows:

1. How prevalent are climate action role model narratives on Instagram and TikTok?
2. What kinds of climate actions do Instagram and TikTok role model narratives focus on?
3. What kinds of calls to action, if any, are included in role model narratives?
4. Are role model narratives correlated with higher engagement (likes) than other types of posts?

## Methodology

### Data collection

Posts that appeared when searching the hashtags #climateaction and #climateactivist were manually collected from Instagram and TikTok using new accounts created for this purpose. #Climateaction was chosen by searching for hashtags including the word “climate” and selecting the most popular hashtag that also included a mention of action-taking, and #climateactivist was chosen as a variation of the #climateaction hashtag that might be more likely to include role models because of its focus on the action-taker. 1,000 posts were collected initially (the first 250 posts per hashtag on each platform). This sample size was chosen to enable in-depth manual content analysis. 355 of these posts were excluded in the analysis phase because the content was no longer accessible or the posts were found not to contain either of the hashtags we searched for (due to Instagram and TikTok’s search algorithms identifying posts that did not contain the hashtags). The posts were collected from 25–30 October 2024, but dated as far back as August 2021. Although this sample does not represent the complete history of the hashtag, it provides a useful snapshot of social media discussion. The final sample breakdown is as follows (Table 1).

**Table 1.** Sample breakdown by hashtag and platform

	#climateaction	#climateactivist	Total
Instagram	238	219	*457*
TikTok	152	36	*188*
*Total*	*390*	*255*	*645*

The following data were manually collected for each post: the URL of the post, the account name, whether the account represented an individual or an organization, the account’s follower count, the date posted, the date accessed, and the number of likes (Instagram posts and TikToks) or views (Instagram Reels).

### Coding

Each post was independently coded by a pair of researchers, and differences were resolved through pair discussion. For decisions of whether each post contained each role model narrative, there was 89% agreement (*α* = 0.527). For decisions of whether each role model narrative contained a call to action, there was 90% agreement (*α* = 0.764). We decided which posts qualified as role model narratives according to the following criteria:

1. Role model narratives must contain a mention of one or more individuals participating in a climate action. Only a photo with no description does not qualify.
2. The behavior modeled must be one that the audience can reasonably perform. For instance, a video about a legislator passing a law or an inventor creating a new product would not count.
3. Narratives depicting climate activists in a critical light (for instance, mocking climate protesters) are not counted as role model narratives.
4. Role model narratives can be about the poster or about someone else, unless the poster is critical of the protagonist of the story. Neutral framings, such as news stories, are included.

For posts that included role model narratives, we inductively coded the type of action modeled by the role model. The list of action types is available in Fig. 3. Secondly, we coded whether each role model post included a specific call to action, defined as instructions to participate in a climate action other than following, commenting, or clicking a link for more information. General calls for action, such as “Let’s make that landslide moment of change together,” were not included. Finally, for role model posts that included calls to action, we inductively coded what type of action was suggested in the call to action (which could be a different type of action than the one the role model was shown engaging in). The list of action types in the calls to action is depicted in Fig. 4.

## Results

### RQ1: Frequency of role model narratives

Across the Instagram posts and TikToks, we found that 13% (83 out of 645) qualified as role model narratives according to our criteria. Other content types included, for instance, discussion of climate news and related political news, advertisements of upcoming events, montages of climate disasters with sorrowful background music, and jokes at the expense of climate activists.

**Figure 1.**
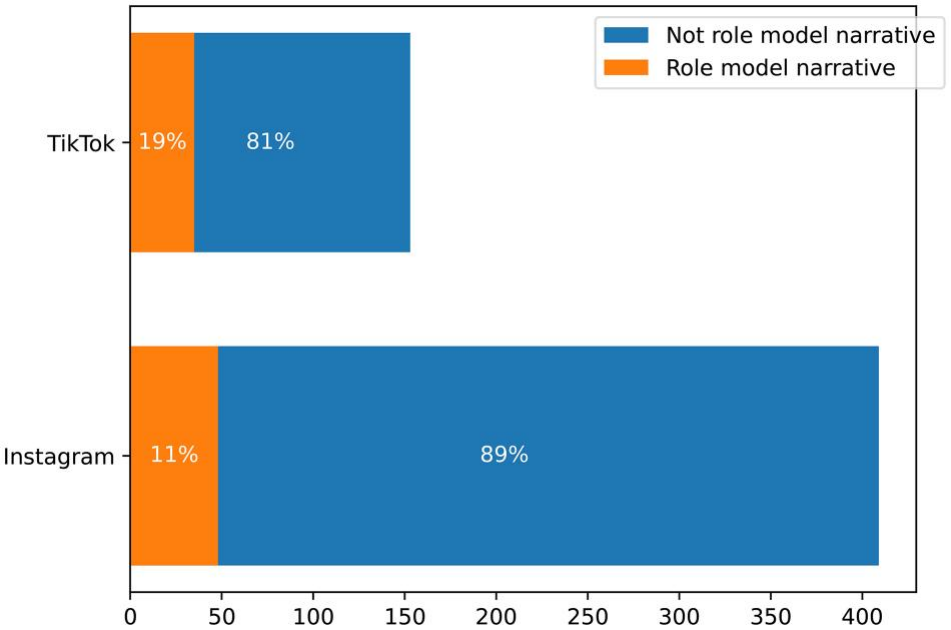
Frequency of role model narratives by platform.

There were significantly more role model narratives on TikTok than on Instagram (*z*-score = −2.797, *P* = 0.005). Nineteen percent of the TikTok posts analyzed were role model narratives, compared to 11% of the Instagram posts (Fig. 1).

**Figure 2.**
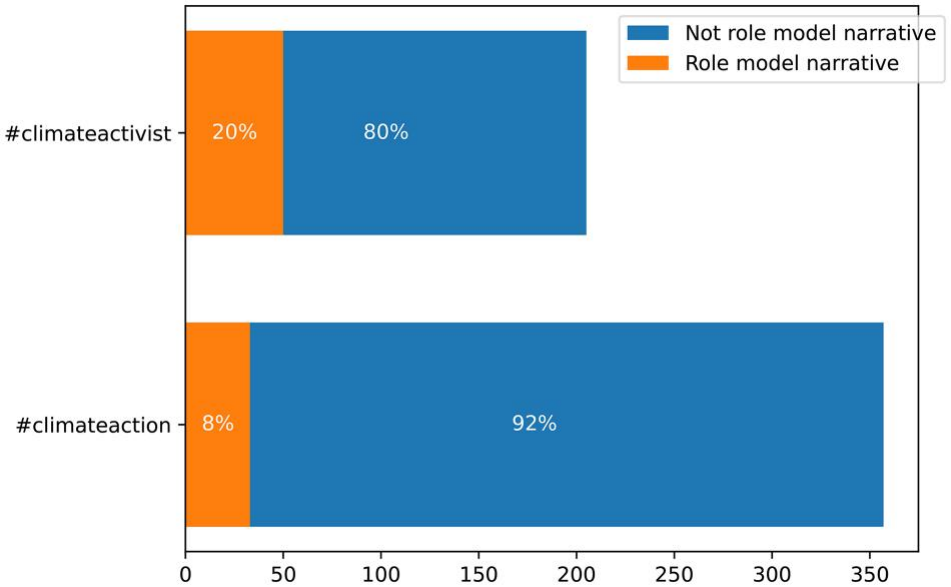
Frequency of role model narratives by hashtag.

Additionally, there were significantly more role model narratives on the hashtag #climateactivist than on #climateaction (*z*-score = −4.133, *P* = 0.000). Twenty percent of the sampled posts tagged with #climateactivist were role model narratives, compared to 8% of the posts tagged with #climateaction (Fig. 2).

**Figure 3.**
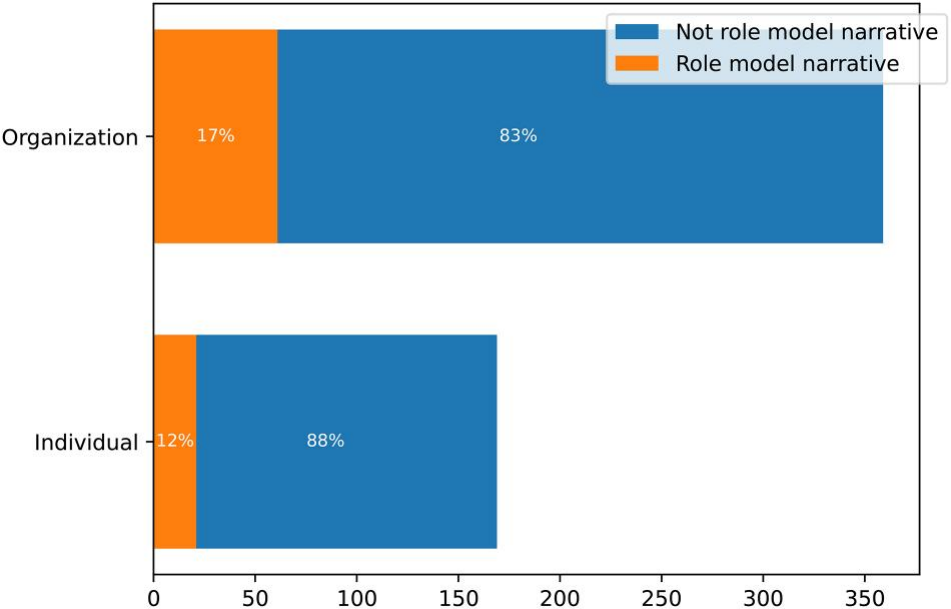
Frequency of role model narratives by account type.

There was no significant difference between the proportion of role model narratives on accounts run by individuals versus organizations (*z*-score = −1.164, *P* = 0.244). Twelve percent of posts by individuals were role model narratives, and 17% of posts by organizations were role model narratives (Fig. 3).

### RQ2: Types of action depicted in role model narratives

**Figure 4.**
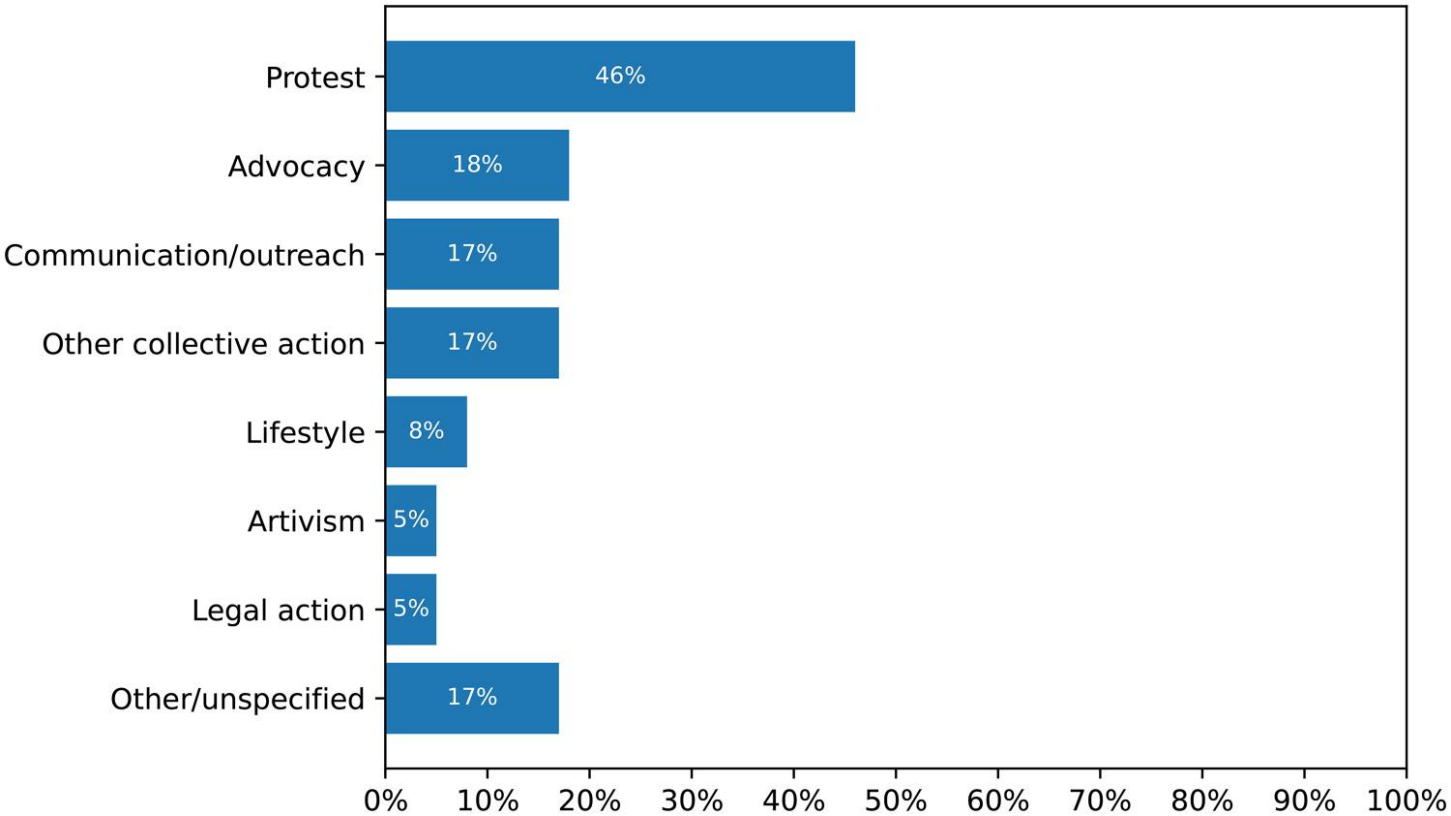
Frequency of types of climate action depicted in role model narratives.

The role model narratives most commonly depicted the role models engaging in protest (46%), such as strikes and marches; advocacy (18%), that is, attempting to persuade decision-makers to act in a way that benefits the climate; communication/outreach to audiences other than decision-makers (17%); other forms of collective action (17%), such as strikes, community organizing, and campaigning; and other climate actions including trash clean-up (4%), tree planting (4%), joining an organization (2%), having a climate job (1%), introspection (1%), invention (1%), job-hunting (1%), voting (1%), and unspecified climate action (1%) (totaling 17%) (Fig. 4).

### RQ3: Calls to action in role model narratives

**Figure 5.**
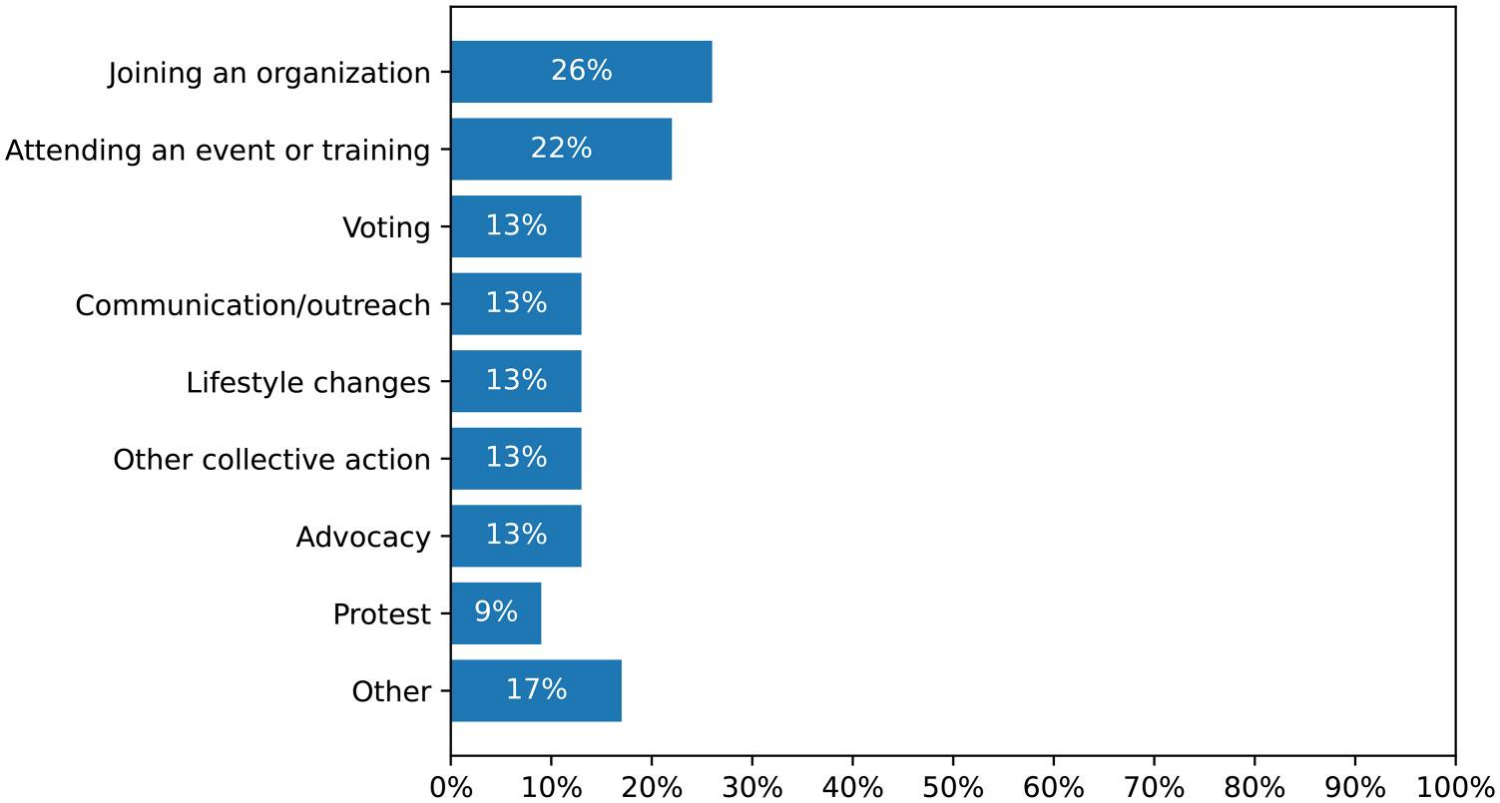
Frequency of types of climate action suggested in calls to action.

Twenty-three of the 83 role model narratives (28%) included calls to action, representing only 4% of the overall sample. Common actions suggested in the calls to action included joining an organization (26%); attending an event or training (22%); voting (13%); communicating about climate change (13%); making lifestyle changes such as switching to LED lightbulbs (13%); participating in other forms of collective action than advocacy, protest, and voting (13%); and engaging in climate advocacy, for instance by signing petitions and emailing legislators (13%) (Fig. 5).

### RQ4: Engagement with role model narratives

We operationalized engagement as the number of likes divided by the number of followers and (due to the skewed distribution) performed a Mann-Whitney *U*-test to evaluate whether role model narratives received more or less engagement than non-role model narratives. There was no significant difference (*U* = 17 884.5, *P* = 0.916). Role model narratives received an average of 0.58 likes per follower, and other posts received an average of 0.91. In addition, of the top 10 best-performing posts in the sample (operationalized as the posts that earned the most likes per follower), two were role model narratives. (In contrast to the overall lower prevalence of posts by individual accounts, four of the 10 top-performing posts were from individuals.)

## Discussion

Despite our expansive definition of role model narratives, their incidence in our sample is low. Because role model narratives are a powerful intervention for encouraging behavior change, this finding suggests that they might be an underutilized communication tool for helping catalyze climate action, at least on social media platforms. While the role model posts in the sample did not generate higher engagement than other kinds of posts, they were represented among the top-performing posts, demonstrating that role model narratives can achieve a broad reach. While reach is not necessarily associated with behavior change, prior research has found that engagement with greenfluencers’ social media posts is associated with activism behaviors [29].

Calls to action have additionally been found to be important for encouraging action, particularly when the actions are pro-social [30, 31]. Most role model narratives in the sample did not include a call to action, indicating that they could potentially be made more effective if they did so. Furthermore, the role model narratives in the sample tended to depict protest (e.g. marches) more so than advocacy (e.g. contacting political representatives). Role model narratives focused on advocacy may be a useful addition since not knowing how to participate in advocacy is one of the most common barriers to participation [32].

Among the few role model narratives that did include calls to action, the types of action depicted tended to be higher involvement than the types of action suggested. For instance, the posts tended to show high-involvement actions such as protesting, but their calls to action generally suggested low-involvement actions such as attending a training. It is possible that there is a need for more role model narratives showing individuals engaging in low-involvement actions, such as signing up for organizations—especially because not knowing how to get involved is a prominent barrier to climate action [33]. However, low-involvement actions might not generate as much engagement as dramatic, high-involvement actions such as protests. Further research could further explore this possible trade-off between generating engagement and matching depicted behaviors to target behaviors.

Additional potential directions for future research include: (1) testing how role model narratives influence climate-related attitudes, beliefs, and behaviors in diverse contexts, including comparing social media to in-person communication; (2) examining the influence of various features of role model narratives, such as the use of first versus third person (see San Cornelio, Martorell, and Ardèvol’s 2024 finding that first-person narratives may be more engaging), as well as the type of behavior depicted [23]; (3) analyzing features of viral climate-related content and exploring whether it is possible to include similar features in role model narratives; (4) understanding the relationship of climate action role model narratives to other environmental role model narratives on social media, such as zero waste influencers [34]; and (5) understanding how to better translate research findings on effective climate communication strategies to communication practice (including on social media) and how to facilitate stronger researcher-practitioner knowledge exchange.

### Limitations

The primary limitations of this study are that we only analyzed posts under two hashtags and on two platforms, over a short time period that was likely influenced by the upcoming US presidential election. We also analyzed a limited set of attributes of each post—for instance, we did not distinguish between first-person and third-person role model narratives. Lastly, given our methodology, it was not possible to track the impacts of each post on the target behavior (collective climate action). Future research could examine target behaviors by surveying users who liked the posts. While we measured post engagement through likes as a way of understanding the performance of posts on social media, post engagement does not necessarily suggest that the posts helped inspire behavior change (which was beyond our methodology’s ability to capture).

## Conclusion

We find that role model narratives about climate action are rare on the social media sites we studied, though they are slightly more common on TikTok and under the #climateactivist hashtag. Furthermore, they tend not to include calls to action, and are predominantly focused on protest more so than advocacy. These results suggest that role model narratives may be an underutilized means of encouraging collective climate action on social media.

## Data Availability

The data underlying this article are available in the article and in its online supplementary material.
